# Repetition of verbal fluency task attenuates the hemodynamic activation in the left prefrontal cortex: Enhancing the clinical usefulness of near-infrared spectroscopy

**DOI:** 10.1371/journal.pone.0193994

**Published:** 2018-03-21

**Authors:** Yoshihiro Kawakubo, Masaya Yanagi, Noa Tsujii, Osamu Shirakawa

**Affiliations:** Department of Neuropsychiatry, Kindai University Faculty of Medicine, Osaka-sayama, Osaka, Japan; Tokai University, JAPAN

## Abstract

In applications of near-infrared spectroscopy (NIRS) in clinical psychiatry settings in Japan, a phonemic verbal fluency test (VFT) that includes “switching” (the ability to shift efficiently to a new word subcategory) to assess phonemic fluency is employed to capture disease-specific hemodynamic changes in the prefrontal cortex (PFC). In this study, to extend the specific features of this test, the VFT was repeated to examine an activation change in NIRS measurements in 20 healthy males. Without task performance change, the hemodynamic activation induced by the VFT was significantly attenuated in the left PFC through repetition of the task. These findings suggest that the left PFC is involved in processing of the VFT. Further, it may be possible to extend the current VFT using this repetition to provide a more sensitive examination of the left PFC, whose dysfunction has been reported in several psychiatric diseases such as major depression, bipolar disorder, and schizophrenia.

## Introduction

On the basis of accumulating evidence regarding near-infrared spectroscopy (NIRS) as a promising simple neuroimaging modality to phenomenologically depict dynamic brain activation, the application of NIRS in psychiatric clinical settings in Japan has been discussed [1, 2]. In a trial in Japan, a phonemic version of the verbal fluency test (VFT) was employed to capture prefrontal hemodynamic changes for the purpose of assisting differential diagnoses for major depressive disorder, bipolar disorder, and schizophrenia [3–6]. The prefrontal activation pattern is characterized in this setting to be small, delayed, and distorted for major depression, bipolar disorder, and schizophrenia, respectively [3–5]. Although this trial has been partially successful in that the phonemic VFT has been approved as a medical examination by the health ministry in Japan, its accuracy remains to be improved, as demonstrated by the fact that the sensitivity of the distinguished prefrontal activation pattern is 52% for major depression (small activation), 66% for bipolar disorder (delayed activation), and 59% for schizophrenia (distorted activation) [7]. Addressing this issue will require an understanding and further development of the current VFT that is qualified for medical examination in Japan, albeit not enough, to assist in the differential diagnosis of these diseases.

VFT consists of two dissociable phenomena of verbal fluency performance: (1) “clustering,” which generates semantically or phonemically related words within subcategories and (2) “switching,” which is the ability to shift efficiently to a new subcategory [8, 9]. Switching is particularly needed for word generation in phonemic VFT, whereas switching and clustering are equally important for semantic VFT [8, 9]. In accordance with these neuropsychological features of the two versions of the VFT, a direct comparison of the two tasks in positron emission tomography (PET) and fMRI studies has shown that phonemic VFT activates the prefrontal cortex (PFC) more strongly than does semantic VFT [10, 11, 12], because switching is primarily subserved by the PFC [8, 9, 13,14].

A unique feature of the phonemic VFT currently used in Japanese clinical psychiatric settings is that it contains switching of designated syllables: three sets of syllables are designated in 20-s segments for each 60-s task period [3–6]. This is a modification of the standard-type phonemic VFT in which the designated syllable is constrained to one syllable for the entire 60-s task period [8]. This modified VFT (m-VFT) may be a task that engages profound switching ability, because structurally, it contains switching of the syllables, and the phonemic VFT principally demands an intense switching ability. These features of the m-VFT may specifically impact frontal cortical activation, which may, to some extent, allow validation of the m-VFT as an assistive device in the differential diagnosis of psychiatric diseases. In this study, to extend the unique feature of this test, the m-VFT was repeated, and the change in hemodynamic activation of the PFC was investigated using NIRS.

## Materials and methods

### Subjects

Twenty healthy males (age: mean ± standard deviation [SD] = 28.2 ± 3.3 years) participated in this study. All were native Japanese speakers and were right handed, as indicated by their Edinburgh Inventory scores [15]. After a complete description of the study was presented, written informed consent was obtained from all subjects. The present study was approved by the Ethics Committee of Kindai University Faculty of Medicine.

### NIRS

A 52-channel NIRS device, ETG-4000 Optical Topography System (Hitachi Medical Co., Tokyo, Japan), was used to measure relative changes in oxy- and deoxy-hemoglobin (Hb) concentrations using two wavelengths (695 and 830 nm) with a sampling rate of 100 ms. Optical data were analyzed with the modified Beer–Lambert law [16] to calculate the signals reflecting changes of Hb levels in arbitrary units (mM–mm). NIRS probes were fixed using 3 × 11 thermoplastic shells, with the lowest probes positioned along the Fp1–Fp2 line according to the International 10–20 system. The distance between pairs of emission and detector probes was set at 3.0 cm, and the measurement area between the probes was defined as a channel. The arrangement of channels covered a portion of the bilateral prefrontal and temporal regions (S1 Fig), where they measured Hb signals of the cortical surface at a depth of 2–3 cm from the scalp [17–19]. This experimental setup was corroborated by a multi-individual study of the anatomical craniocerebral correction using the International 10–20 system [20–22].

NIRS data analysis was performed according to the manual for clinical psychiatric settings in Japan for NIRS measurements, as shown in previous reports [3–6, 23]. Briefly, relative changes of oxy-Hb data were analyzed to estimate regional brain activation, and the mean oxy-Hb signals during the task period were analyzed using the “integral mode”: the pre-task baseline level was defined as the mean oxy-Hb measured for 10 s immediately before the task period, the post-task baseline level was defined as the mean oxy-Hb for the last 5 s in the post-task period, and linear fitting was then applied to the data between these pre- and post-task baseline levels [3, 4, 23]. A 5-s moving average was calculated to eliminate any short-term motion artifacts. Based on previous NIRS studies, the data acquired from the 21 channels in the upper two rows were excluded from statistical analysis, because they clearly contained artifacts, presumably because of the presence of hair and also because their signal-to-noise ratio seemed to be low or approaching zero [5, 6, 23].

### Cognitive task

**Fig 1:**
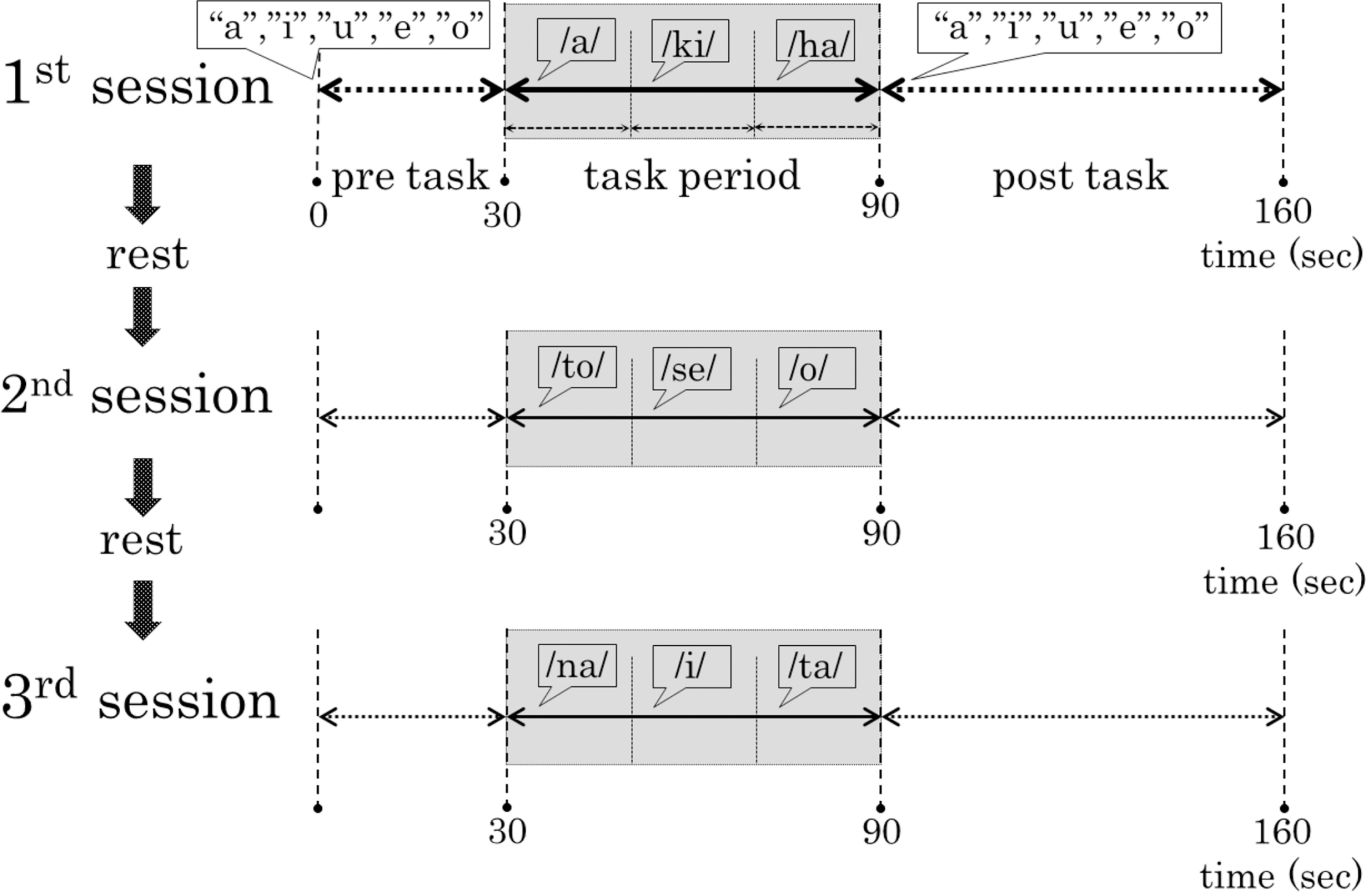
Design of the m-VFT set.

NIRS measurements were conducted using a series of three m-VFT sessions. Each m-VFT session was performed according to previous reports [3–6], and included a 30-s pre-task baseline period, a 60-s task period comprising three 20-s blocks, and a 70-s post-task baseline period (Fig 1). Subjects were instructed to repeat a sequence of syllables (a, i, u, e, o) during the pre-task and post-task baseline periods. During the task, subjects were asked to generate as many words as possible starting with the presented syllable on a computer display placed in front of them. Generated words were marked as either correct or incorrect; the number of correct words represented the subject’s performance score in the task. As Fig 1 shows, a series of three sessions of the m-VFT were repeated, switching the designated syllables to avoid repetition of the same stimuli. One of the three syllables was assigned to block 1 (seconds 0–20): /a/, /to/, or /na/; block 2 (seconds 20–40): /i/, /ki/, or /se/; and block 3 (seconds 40–60): /o/, /ta/, or /ha/. Three-min rest intervals were taken between the m-VFT sessions. Throughout the three m-VFT sessions, NIRS probes were not removed to avoid changing the optical path length of the probes.

### Data analysis

Performance scores were compared among the three m-VFT sessions using repeated measures analysis of variance (ANOVA) with the Greenhouse-Geisser correction. Similarly, mean oxy-Hb signals were compared among the three m-VFT sessions across all subjects using repeated-measures ANOVA with the Greenhouse-Geisser correction. If this ANOVA yielded significance, Dunnett’s post-hoc multiple comparison test was conducted to detect a significant change in the second or third compared with the first m-VFT session. Because we examined a total of 31 channels (referred to as “ch22-52” throughout) in the lower three rows, the threshold for significance of *p*-values was set at 0.0016 (0.05/31) using the Bonferroni correction. All statistical tests were performed with GraphPad Prism 6.0 for Windows version 6.07 (GraphPad Software, Inc., La Jolla, CA, USA).

## Results

### Task performance

The number of words generated during the task did not differ significantly among the three m-VFT sessions (first session: mean ± SD = 15.7 ± 3.5, second session: 15.8 ± 3.7, and third session: 16.8 ± 3.3; *F* [1.8, 35.0] = 1.3, *p* = 0.29).

### NIRS data analysis

The m-VFT, especially the 1^st^ session, revealed increased oxy-Hb signals during the task in all the NIRS channels analyzed in this study. Within the 31 channels, those judged by ETG-4000 software to be probe errors throughout the three m-VFT sessions were excluded from the analysis. According to this criterion, ch42 was excluded in two subjects, and ch23, 26, 31, 32, 33, 37, and 51 were excluded in one subject.

**Fig 2:**
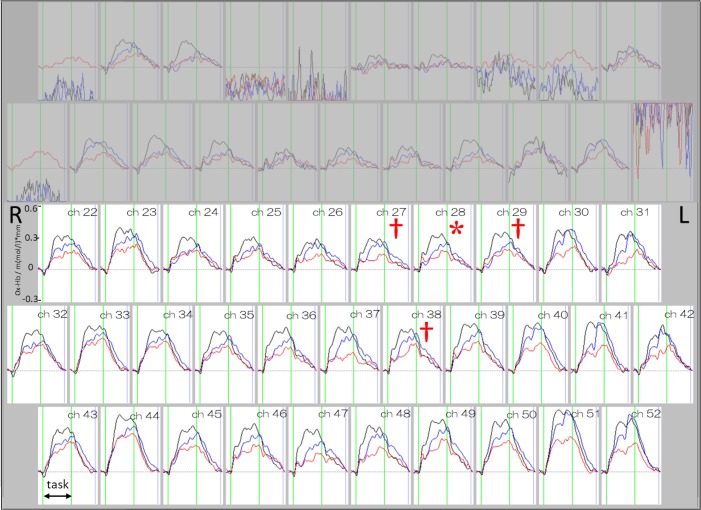
Grand averages of waveforms of oxy-Hb signal changes across all subjects in three m-VFT sessions (black line: First session, blue line: Second session, and red line: Third session). † represents *p* < 0.0016 in ANOVA. * represents *p* < 0.0016 in ANOVA with post-hoc test (first vs third session). Bonferroni-corrected *α*-level was 0.0016 (0.05/31).

**Fig 3:**
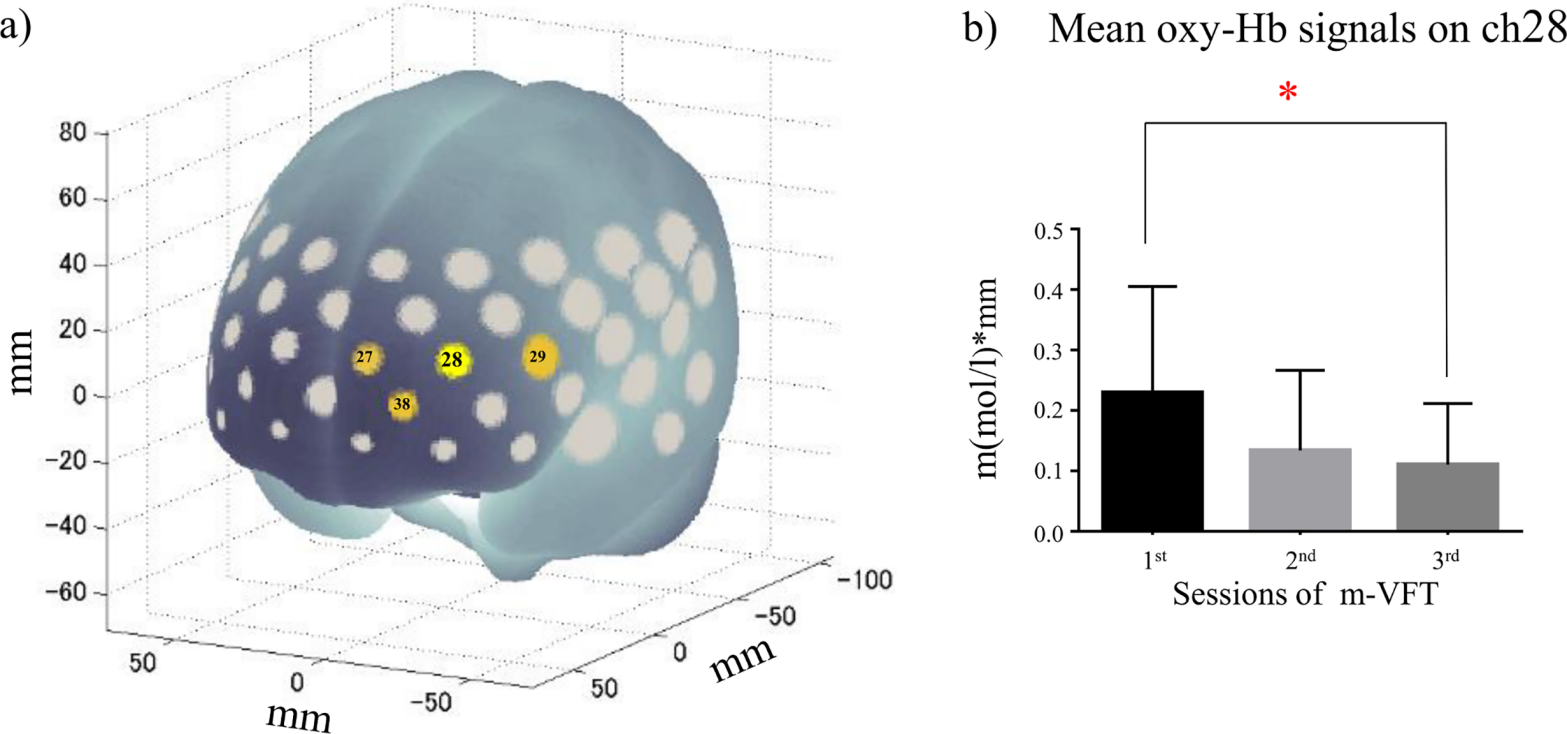
(a) Location of channels where task-induced activation was significantly affected by repetition of m-VFT (highlighted in yellow). (b) Mean oxy-Hb signals during the task in three m-VFT sessions on ch28. Significant attenuation of the activation was detected between first and third sessions on this channel.

In the repeated-measures ANOVA examining mean oxy-Hb signals during the task, significant differences were found on ch27 (*F*[DFn = 1.7, DFd = 31.7] = 9.0, *p* = 0.0014), ch28 (*F*[1.7, 31.5] = 9.7, *p* = 0.0010), ch29 (*F*[1.5, 28.7] = 9.9, *p* = 0.0013), and ch38 (*F*[1.9, 35.2] = 8.7, *p* = 0.0011; Figs 2 and 3A). All *p*-values in the repeated-measures ANOVA for the remaining channels were above 0.0016. Dunnett’s post-hoc multiple comparison test of the four channels showed that mean oxy-Hb signals during the task were significantly decreased in the third compared with the first session on ch28 (first vs second session: *p* = 0.0206; first vs third session: *p* = 0.0008; Fig 3B). No significant differences in mean oxy-Hb signals during the task were observed on ch27 (first vs second session: *p* = 0.0353; first vs third session: *p* = 0.0035), ch29 (first vs second session: *p* = 0.0073; first vs third session: *p* = 0.0046), or ch38 (first vs second session: *p* = 0.0612; first vs third session: *p* = 0.0027).

Please refer to S1 Table. for the details of analyzed data.

## Discussion

The present study was the first NIRS study, to our knowledge, to show how phonemic VFT-induced regional brain activation was changed during repetitive VFTs. Without obvious task performance change, which ensures the consistent commitment of subjects to the task, hemodynamic activation was significantly attenuated by repeating the m-VFT on four channels: ch27, 28, 29, and 38. These four channels were located in the left PFC. In contrast; none of the channels located in the right hemisphere reached statistical significance. The attenuation of m-VFT-induced activation was pronounced on ch28, which is annotated in the left dorsolateral PFC (DLPFC) according to a virtual registration of the NIRS channels [20–22, 24]. These findings demonstrate that repetition of the m-VFT attenuated the task-induced activation in the left PFC, where the attenuation was presumably emphasized in the left DLPFC.

The left PFC is a central region for verbal processing [25, 26]. Within the PFC, working memory postulates a crucial role in the dorsolateral portion [27, 28]. Consistent with this line of evidence, a PET study showed that the left DLPFC mediates the manipulation of verbal working memory [29]. Several fMRI studies reported that working memory tasks induced less activation after repetition of the task [30, 31], even without obvious task performance change [32, 33]. One mechanism underlying this phenomenon is believed to be improved neural efficiency of task processing [30, 32–35]. Because the automated processing induced by repeating a working memory task results in a reduced demand on the working memory load, it also reduces the activity in the task-related brain areas such as left DLPFC [31]. Combined with the evidence that working memory is engaged in the VFT [36, 37], these lines of evidence suggest that our results—that is, the attenuated activation around the left DLPFC without task performance change—are due to an improved neural efficiency to manipulate the VFT. Nonetheless, some other possibilities cannot be excluded, which may potentially explain the decrease of the oxy-Hb found in our study, e.g., mediation of some cognitive process may be implemented only in the initial phase of task learning [32] or over-activation amplified in the initial phase of task may be optimized during task repetition [38].

A number of fMRI studies have shown that the left PFC is potently activated by the phonemic VFT [39, 40], and a fMRI study reported that a phonemic VFT that contained switching of the designated syllables exhibited left PFC hypoactivation in depressed patients compared with controls [41]. While NIRS studies show bilateral fronto-temporal activation during the m-VFT in healthy controls [4–6], depressed patients are reported to have a weaker activation in the left PFC during the first half of the task [42]. Because the laterality change was found in the VFT with the switching of the designated syllables in these studies, Klumpp et al. hypothesized that VFTs that require more switching among phonemes may be more sensitive to left frontal lobe deficits than those with less switching [43]. Our results support this hypothesis from the viewpoint of normal engagement of the left PFC in manipulating the VFT. Further, our results could guide further development of the current VFT to focus more on left PFC dysfunction. Namely, repetition of the m-VFT could provide a more sensitive examination of left PFC function in terms of the efficiency of neural processing, in addition to observations from the single m-VFT that is currently used in clinical psychiatry settings in Japan. This enriched information from repeating m-VFT may be useful even for classifying a disease into subtypes that could be associated with some specific aspects of the disease, such as a symptom or treatment response.

This study had a few methodological limitations. First, the spatial resolution of NIRS was insufficient to precisely annotate the channel locations on the segmentalized cortical surface area. A study using fMRI in conjunction with NIRS would be valuable in future analysis to validate the exact brain areas where the NIRS channels detected altered activation in this study. Second, a certain portion of oxy-Hb signals acquired by the NIRS device contains some local skin blood flow [44, 45]; therefore, we cannot exclude the possibility that the attenuation of m-VFT-induced hemodynamic activation was not fully derived from the brain reaction. Nevertheless, based on our results, we must remember that when the VFT is applied in clinical assessments, repeating the VFT (even when switching the designated syllables) attenuates the task-induced hemodynamic activation on some of the frontal channels in NIRS measurements.

In this study, we found that repetition of the m-VFT attenuated left PFC activation in NIRS measurements. This finding suggests that left PFC is a sensitive region for processing the m-VFT. This would support Klumpp’s hypothesis, which points out a possible association between the switching in VFT and sensitivity of the left frontal dysfunction [43]. Taken together, these results indicate that repetition of the m-VFT may enhance the current, single-use m-VFT by focusing more on the left PFC, whose dysfunction has been reported in several psychiatric diseases such as major depressive disorder, bipolar disorder, and schizophrenia [43, 46, 47]. Further studies should examine the task-induced hemodynamic changes in psychiatric diseases, from the viewpoint of their particular pathophysiologies.

## Supporting information

S1 Table Details of analyzed data. (XLSX)

S1 Fig Channel locations for near-infrared spectroscopy. (TIF)
