# Supplementary figures and images for: Repetition of verbal fluency task attenuates the hemodynamic activation in the left prefrontal cortex: Enhancing the clinical usefulness of near-infrared spectroscopy
Source: PLoS One. 2018 Mar 21;13(3):e0193994. doi: 10.1371/journal.pone.0193994 (PMC5862477; doi:10.1371/journal.pone.0193994)

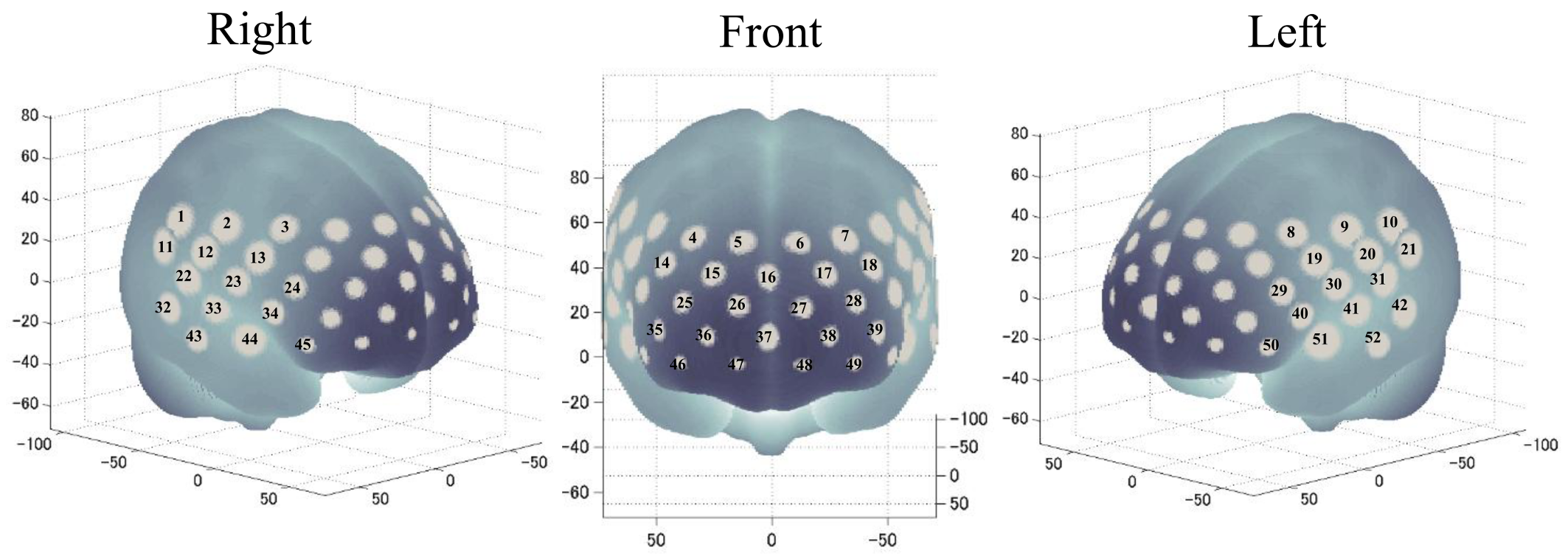

Supplement: S1 Fig — (TIF) [file pone.0193994.s002.tif]
